# Urbs in Rure

**Published:** 1910-11-19

**Authors:** 


### The Hospital

A Journal of

The Medical Sciences and Hospital Administration.

New Series. No. 197, Vol. VIII. [No. 1269, Vol. XLIX.] SATURDAY, NOVEMBER 19, 1910.

### Urbs in Rure.

Besides the continual growth of suburbs and the garden city and "back-to-the-land" movements, there are other ways in which relief has been found from the increasing urbanisation of this country. The first great step was taken when State-maintained asylums for the insane came into existence. Reformatories, epileptic colonies, modern penal institutions, convalescent homes, and open-air schools, are further examples of what of late years has been found increasingly necessary in the way of providing rural or secluded surroundings for various sections of the community. About the latest development of the same principle of *urbs in rure* is the sanatorium.

What is at present chiefly noticeable (to the observant person) about sanatoriums is that although they come in, often quite rightly perhaps, for plenty of adverse criticism, yet nearly every week there is mooted the establishment of a fresh one. And they are, indeed, giving a strong impetus to the spread of the principle just mentioned. It is not hard to see that town-supported institutions will come in time to exercise a powerful modifying effect on English country life. To put the case more plainly, the new hundred or fifty bed building is a much better local customer than ever the hall or parsonage can be. This fact, if interesting, is however a little beside the central point of these remarks. To return to sanatoriums, it has been found that the great weakness of their system of therapeutics is the liability of a considerable proportion of patients to relapse after their discharge back into a world which is far from being specially arranged to suit the health of those convalescent from pulmonary tuberculosis. This fact was realised pretty early, and efforts were made to cope with it. Sanatoriums were cheapened in construction in order to save as much money as possible for maintenance. Dr. Paterson, of Frimley, proved that consumptives could be trained to work out some part of their cost, if not exactly to their benefit, speaking absolutely, yet with advantage to themselves, considering their inevitable return to occupations often arduous. Best of all, the idea of the labour colony fed by the sanatorium was mooted, although it must be admitted that this proposed development cannot be judged on results—because as yet such institutions do not exist, apart from that just started near Edinburgh—or from mere approximations such as that made by Dr. Chapman, and such as, we are glad to learn, the Kelling Sanatorium authorities are promoting in Norfolk.

This latter experiment was described at the Conference on the employment of consumptives arranged by the Charity Organisation Society, and fully reported in our issue of last week. Those present seemed agreed that patients ought to have careful watching for two years after discharge, preferably in a labour colony, since ordinary open-air after-occupation was of itself no security for recovery. It must be remembered in connection with this latter statement that in the current labour market outdoor employment mostly calls for severe and sustained physical exertion, a demand which is not likely in the long run to be well met by the spare muscles of the typical consumptive. In open competition, too, there is no provision for an occasional day or week in bed followed by a change temporarily to lighter occupation, such as may make all the difference, in the case of our convalescent, between sinking and swimming, so to say. And with the patient under no medical supervision except such as he chooses to summon at pecuniary cost to himself, he can much more easily neglect to look after himself properly than if his employer were also his physician. All these are obviously points where should come in the labour colony conducted as a supplement to and in connection with the sanatorium. The various kinds of estate work, ranging from hoeing weeds in a garden to draining work and tree felling, from driving cattle to pasture to filling a coal cart, from painting to digging, from poultry feeding to mason's work, furnish a multitude of tasks fitted for every grade of physical fitness, and, at the same time, supply some financial basis for the experiment in reducing the food bills and cost of upkeep of the sanatorium itself. Naturally there is needed devoted personal attention from a medical superintendent, since there is no money to spare for the salary of the official known at asylums as the steward. But some such plan as this all sanatoriums will probably have to adopt before long, if only in self-defence—namely, to prevent patients relapsing soon after discharge, and to take tuberculous unemployables off the hands of Boards of Guardians, who will grow more willing to send patients for treatment if they see hopeful cases definitely provided for. It naturally involves, as has been seen, that medical officers and others should make themselves acquainted with the elements of land agency work, and with some of that multifarious knowledge possessed by the individual known as a " clerk of works." But in practice this should prove no serious obstacle to the natural development of centres of active anti-tuberculosis effort, effort of a kind particularly valuable and prepossessing, and yet at present hardly to be found.

